# The Effects of Different Oil Sources on Performance, Digestive Enzymes, Carcass Traits, Biochemical, Immunological, Antioxidant, and Morphometric Responses of Broiler Chicks

**DOI:** 10.3389/fvets.2020.00181

**Published:** 2020-04-28

**Authors:** Youssef A. Attia, Mohammed A. Al-Harthi, Hayam M. Abo El-Maaty

**Affiliations:** ^1^Arid Land Agriculture Department, Faculty of Meteorology, Environment, and Arid Land Agriculture, King Abdulaziz University, Jeddah, Saudi Arabia; ^2^Poultry Production Department, Faculty of Agriculture, Mansoura University, Mansoura, Egypt

**Keywords:** fish oil, coconut oil, canola oil, broiler performance, immunological response

## Abstract

This research evaluate the influence of different oil sources, namely fish oil (FO), coconut oil (CocO), canola oil (CanO), or a mixture of the three oils (MTO)—included at 1.5% in broiler diets—compared to a no oil-supplemented diet. Hence, 250 unsexed, 1-day-old Cobb chicks were weighed and randomly allocated into five dietary treatment groups of 50 chicks each and five replicates per group. Oil-supplemented diets significantly increased the growth, improved the feed conversion ratio (FCR), and decreased the abdominal fat percentage compared to the control diet. Amylase was significantly elevated due to feeding the FO- or CocO-supplemented-diet compared to the control diet, whereas lipase increased due to offering CocO- and CanO-enriched diet; chymotrypsin increased due to different oil sources. High-density lipoprotein cholesterol (HDL-C) increased markedly due to offering an oil-supplemented diet, but low-density lipoprotein cholesterol (LDL-C), the LDL-C:HDL-C ratio, and malondialdehyde (MDA) decreased. Blood plasma immunoglobulin (Ig) G and IgM significantly increased due to feeding CocO, CanO, or MTO compared to the control group, whereas FO increased IgG only. FO- and CanO-containing diets resulted in the highest increase in α2-globulin and γ-globulin. The antibody titer to avian influenza (HIAI) and Newcastle disease (HIND) were significantly elevated due to CocO supplementation compared to the control group. The bursa follicle length and width and thymus cortex depth were increased considerably due to the FO-supplemented diet compared to the control, but the follicle length:width ratio decreased. The villus height:depth ratio was significantly elevated due to both the CanO and MTO diets. The antioxidant status improved considerably due to the addition of CocO and CanO. Both CanO and MTO similarly increased plasma T3, T4, and the T3:T4 ratio. In conclusion, oil supplementations at 1.5% enhanced growth performance and immune status, improved the blood lipid profile and antioxidants status, and the effect of the oil sources depends on the criteria of response.

## Introduction

Poultry is a very important industry. Indeed, it is estimated that in 2020, chicken will be the most consumed animal protein in the world. Fat and oil are generally used in poultry diets to increase the energy concentration. Fat-enhanced feeds increase the efficiency of the feed energy and productivity in poultry (1, 2). Moreover, oil improves the absorption of fat-soluble vitamins, the palatability of diets, decreases the dustiness of feeds, and reduces the passage rate of feed in the gut, which provides more time for the sufficient absorption of nutrients (3–5). Besides, the fatty acid profile of muscle tissue mirrors the dietary lipid profile and can alter the blood levels of lipoproteins and triglycerides (6–8). As a rule, the utilization of unsaturated fatty acids (UFAs) in poultry diets improves the product quality (for example, ω3 and ω6). This improvement is concordant with the consumers' interest (9, 10) and immune response (3, 4, 11–13).

Fish oil (FO) has high percentages of long-chain polyunsaturated fatty acids (PUFA), particularly ω3 fatty acids that increase oxidative damage and negatively affect the flavor of animal products. FO is low in ω6 fatty acids and linoleic acid (14, 15). Two types of ω3 fatty acids are found in fish meal: eicosapentaenoic acid (EPA) and docosahexaenoic acid (DHA) (15). Alpha-linolenic acid (ALA) is found in plant seeds and can be transformed to EPA and DHA in the body (16). Corn oil, animal fat, animal and vegetable fat blends, and FO do not affect the feed consumption but improve the growth rate and feed conversion rate (FCR) in broilers exposed to heat stress compared to un-supplemented diets with heat stress (17).

Dietary ω3 PUFA sources, such as FO or linseed oil, have a positive significant impact on humoral immunity, i.e., antibody titers against the Newcastle disease virus (NDV), compared to the control diet (18). FO has no negative effects on the immune function of broilers (19). Furthermore, FO significantly increases antibody titers and the relative weight percentages of the bursa and spleen compared to a control diet (20). Birds fed a diet with an ω6: ω3 ratio that exceeds the recommended levels show damages to the intestinal epithelial cells (21). Further, a low ω6: ω3 ratio increases malondialdehyde (MDA) in tissues, including the muscle, and thus impairs meat quality.

Less than 2% of erucic acid (docosenoic acid, C22:1, ω-9) of rapeseed variety is called canola oil (CanO) (22). High erucic acid levels in the diet harm the growth, feed intake, and digestibility of lipids (23). Furthermore, chicks fed diets that contain erucic acid deposit less fat and use lipid energy less efficiently (23). Feeding two varieties of CanO to female broilers increases the growth rate compared to feeding tallow and acidulated soybean oil soap stock. These data confirm the advantages of using CanO as energy sources for birds (24). The better growth rates are a result of the higher percentage of long-chain fatty acids (LCFA) and high triglyceride content (24). Broilers fed a diet with poultry fat, CanO, sunflower oil, corn oil, soybean oil, or lard exhibit similar growth performance as well as cuts and carcass yields at day 49 of age (25). Broilers fed corn oil and lard show higher red-colored meat compared to those fed on CanO, soybean oil, and sunflower oil, but the difference from the poultry fat is not significant (26).

Coconut oil (CO) is a rich source of saturated fatty acids (SFA); they comprise ~90% of the total fatty acid content. Medium-chain fatty acids (MCFA, C6-C12) represent ~60% of the entire fatty acid content (27). These fatty acids are absorbed directly into the portal circulation without re-esterification in the intestinal cells (28). MCFA are burned exclusively and rapidly for energy production (29). In contrast, the LCFA (28) are deposited in the adipose tissue (30). MCFA reportedly decrease fat deposition in the meat (31–33) and enhance blood lipid profiles in humans (34) and rats (31). However, experiments with broilers indicated that MCFA reduces the growth rate (35). Intriguingly, CocO enhances fat digestion and growth performance of broilers during a coccidiosis infection (36).

Currently, there is considerable interest in producing functional food and/or value-added products such as eggs, meat and milk (8). The sources of ω3 fatty acids are among the most exciting potential applications in animal products due to their health benefits (37, 38). Meat quality and fatty acids are strongly affected by dietary fat/oil sources (8, 10, 39–41) and might significantly reduce the atherogenic, thrombogenic, and cholesterol effects of animal products (37, 38). Although some information is available on the effect of the use of individual oils on broiler performance, blood lipid profile, and immunity (8, 42), the results on the impact of the combination of different oil sources on the production, pancreatic enzymes, meat quality, metabolic profiles, and immune status in broilers are limited. Therefore, this study evaluated the effect of feeding a diet that contained 1.5% of FO, CocO, CanO, or a mixture of the three oils (MTO) on the performance, digestive enzymes, carcass traits, biochemical, immunological, antioxidant, and morphometry responses of broilers chickens.

## Materials and Methods

The experimental protocol was approved by the scientific committee of the Poultry Production Department, Faculty of Agriculture, Mansoura University. The care and handling of the animals were performed to maintain their rights, ensure their welfare, and cause minimal stress, according to International Guidelines for research involving animals (Directive2010/63/EU).

### Experimental Design, Animals, Diets, and Management

A total of 200 unsexed, 1-day-old Cobb chicks were weighed and equally divided among four experimental groups (50 chicks each). The chicks in each group were subdivided into five equal replicates (10 chicks per replicate), and each replicate was housed in 1 × 1 m floor pens in open-sides house furnished with wood shaving for litter. Each pen was equipped with a tube pen feeder and 5-L waterer. The first, second, third, and fourth groups were fed a diet with 1.5% FO, CocO, CanO, or MTO (FO + CocO + CanO of 0.5% each), respectively. The oils were added to the basal diet to isocaloric and isonitrogenous feeds to minimize the interaction between dietary and supplemented fats.

The experimental starter and growth basal diets were composed mainly of maize, soybean meal, and corn gluten meal and formulated using feedstuff composition recommended by the National Research Council (43). The experimental diets met the Cobb breeding guide. The ingredient composition and calculated analysis of the basal diets are shown in Tables 1, 2. The chicks were offered a starter diet—containing 23% crude protein and ~13.2 MJ/kg metabolizable energy (ME)—up to 21 days of age. Subsequently, they were switched to the growth diets, which contained ~20% CP and 13.2 MJ/kg ME, from 21 to 42 days of age.

**Table 1:** The calculated composition and chemical analyses of the experimental diets fed to broiler chicks from 0 to 21 weeks of age.

**Ingredients, %**	**FO^1^-diet**	**CocO^2^-diet**	**CanO^3^-diet**	**MTO^4^-diet**
Ground yellow corn	60.1	60.1	60.1	60.1
Soybean meal 44%	18.0	18.0	18.0	18.0
Corn gluten meal 60%	15.5	15.5	15.5	15.5
Fish oil (FO)	1.50	–	–	0.50
Coconut oil (CocO)	–	1.50	–	0.50
Canola oil (CanO)	–	–	1.50	0.50
Ground limestone	2.0	2.0	2.0	2.0
Dicalcium phosphate	1.8	1.8	1.8	1.8
Vitamin and mineral Premix^5^	0.30	0.30	0.30	0.30
Sodium chloride	0.30	0.30	0.30	0.30
DL-Methionine	–	–	–	–
L-Lysine-HCl	0.50	0.50	0.50	0.50
Total	100	100	100	100
**Calculated analysis (as fed basis)**				
Metabolizable energy MJ/kg	13.2	13.1	13.1	13.1
Crude protein %	23.1	23.1	23.1	23.1
Ether extract %	2.82	2.82	2.82	2.82
Crude fiber %	2.78	2.78	2.78	2.78
Calcium %	1.20	1.20	1.20	1.20
Nonphytate P %	0.451	0.453	0.452	0.451
Lysine %	1.19	1.19	1.19	1.19
Methionine %	0.453	0.451	0.452	0.453
Methionine + Cystine %	0.852	0.853	0.851	0.851
**Determined analysis (as fed basis)**				
Dry matter %	89.0	89.1	88.9	88.9
Organic matter %	79.1	80.1	79.8	79.9
Crude protein %	23.0	22.9	23.0	23.0
Ether extract %	3.17	3.16	3.17	3.17
Crude fiber %	3.12	3.11	3.13	3.13
Ash %	9.89	9.05	9.13	9.07
Nitrogen-free extract %	46.9	47.9	47.6	47.6

1 FO, Fish oil;

2 CocO, Coconut oil;

3 CanO, Canola oil;

4 MTO, Mixture of three oilds (FO + CocO + CanO);

5 *Each 3 kg of preMTO contained: vit. A 12,000,000 IU, vit. D3 3,500,000 IU, vit. E 20 g, vit. K3 3 g, vit. B1 3 g, vit. B2 8 g, vit. B6 3 g, vit. B12 15 mg, Ca pantothenate 12 g, Niacin 40 g, Folic acid 1.5 g, Biotin 50 mg, Choline chloride 600 g, Mn 80 g, Zn 75 g, Fe 40 g, Cu 10 g, I 2 g, Se 0.3 g, Co 0.25 g, and CaCo3 as a carrier*.

**Table 2:** The calculated composition and chemical analyses of the experimental diets fed to broiler chicks from 21 to 42 weeks of age.

**Ingredients, %**	**FO^1^-diet**	**CocO^2^-diet**	**CanO^3^-diet**	**MTO^4^-diet**
Ground yellow corn	66.35	66.35	66.35	66.35
Soybean meal 44%	16.0	16.0	16.0	16.0
Corn gluten meal 60%	11.5	11.5	11.5	11.5
Fish oil (FO)	1.5	–	–	0.50
Coconut oil (CocO)	–	1.5	–	0.50
Canola oil (CanO)	–	–	1.5	0.50
Ground limestone	2.0	2.0	2.0	2.0
Dicalcium phosphate	1.8	1.8	1.8	1.8
Vitamin and mineral Premix^5^	0.30	0.30	0.30	0.30
Sodium chloride	0.30	0.30	0.30	0.30
DL-Methionine	–	–	–	–
L-Lysine-HCl	0.25	0.25	0.25	0.25
Total	100	100	100	100
**Calculated analysis (as fed basis)**				
Metabolizable energy MJ/kg	13.2	13.2	31.2	13.2
Crude protein %	20.0	20.0	20.0	20.0
Ether extract %	2.94	2.94	2.94	2.94
Crude fiber %	2.73	2.73	2.73	2.73
Calcium %	1.19	1.19	1.19	1.19
Nonphytate P %	0.45	0.45	0.45	0.45
Lysine %	0.922	0.921	0.923	0.922
Methionine %	0.392	0.391	0.392	0.391
Methionine + Cystine %	0.743	0.742	0.741	0.741
**Determined analysis (as fed basis)**				
Dry matter %	91.0	90.9	90.9	90.7
Organic matter %	81.7	81.7	81.7	81.5
Crude protein %	22.0	22.0	22.0	22.1
Ether extract %	3.23	3.23	3.20	3.24
Crude fiber %	3.00	3.03	2.99	3.01
Ash %	9.26	9.17	9.21	9.18
Nitrogen-free extract %	53.5	52.5	53.5	53.1

1 FO, Fish oil;

2 CocO, Coconut oil;

3 CanO, Canola oil;

4 *MTO, Mixture of three oilds (FO + CocO + CanO)*.

5 *Each 3 kg of preMTO contained: vit. A 12,000,000 IU, vit. D3 3,500,000 IU, vit. E 20 g, vit. K3 3 g, vit. B1 3 g, vit. B2 8 g, vit. B6 3 g, vit. B12 15 mg, Ca pantothenate 12 g, Niacin 40 g, Folic acid 1.5 g, Biotin 50 mg, Choline chloride 600 g, Mn 80 g, Zn 75 g, Fe 40 g, Cu 10 g, I 2 g, Se 0.3 g, Co 0.25 g, and CaCo3 as a carrier*.

All chicks were kept under similar managerial, hygienic, and environmental. The vaccination and medical care program were carried out under the supervision of a veterinarian. In brief, the birds were vaccinated against IB+ NDV (Hitchner B1 strain) at day 8, Gumboro at day 15, and the NDV LaSota strain at day 21. Feed and water were offered *ad libitum*. Broilers were provided with a 23 h light:1 h dark photoperiod. The indoor temperature and relative humidity (RH) during the experimental period were 34°C and 43%, 32°C and 45%, and 30°C and 51% during weeks 1, 2, and 3 of age, respectively. The ambient temperature and RH during weeks 4, 5, and 6 were 29.7°C and 55%, 30.2°C and 56%, and 30.8°C and 55%, respectively.

### Criteria of Response

The body weight and feed intake of each replicate were recorded at days 1, 21, and 42 of age. Records of live body weights (LBW) and feed intake (FI) were used to calculate the FCR, and (44) for the European production index.

At 42 days of age, five birds from each treatment were randomly selected to represent all treatment replicates; each selected bird was around the average body weight for the group. These specimens were weighed, fasted overnight, and then slaughtered according to a previously reported Islamic method (45). After complete bleeding, the feathers were plucked, the carcasses eviscerated, and hot carcasses were weighed. The weight of the abdominal fat, liver, gizzard, heart, giblets (liver + gizzard + heart), and total edible parts (hot carcass + giblets) were recorded and expressed as a ratio to live body weight.

Five blood samples per treatment—representative of all replicates—were collected from the brachial vein with a vacutainer tube using heparinized and non-heparinized tubes at day 29 to determine the serum antibody titer and at day 42 to determine the biochemical constituents of the blood. Serum and plasma were separated by centrifugation at 1,500 g for 15 min. The blood samples used for analyses were collected before the start of all vaccinations (day 0) and after the end of the last vaccination (day 29). Total antibody production specific for the NDV vaccine was determined in serum using commercial enzyme-linked immunosorbent assay (ELISA) kits (46). The antibody response was measured by the hemagglutination inhibition (HI) test (47). The assay is designed to measure IBD antibody bound to influenza antigen-coated plates (48). The Takatsy method (49) was used to determine the HI against NDV, avian influenza (AI), and infectious bronchitis disease (IBB).

The biochemical markers were assayed using commercial diagnostic kits (Spectrum Diagnostics, Obour City, Egypt) unless otherwise stated. The total protein, albumin, triglyceride, total cholesterol, and high-density lipoprotein cholesterol (HDL-C) levels in the blood plasma were measured at day 42 of age. The level of low-density lipoprotein cholesterol (LDL-C) in blood plasma was estimated (50) as follows: LDL-C = Total Cholesterol–(HDL-C + VLDL); where very-low-density lipoprotein (VLDL) was estimated as the concentration of plasma triglycerides divided by five. In addition, the activities of plasma superoxide dismutase (SOD), catalase (CAT), malondialdehyde (MDA), alanine aminotransferase (ALT), and aspartate aminotransferase (AST) as well as the total antioxidant capacity (TAC) were determined. Alkaline phosphatase (AlkP), immunoglobulin (Ig)G, IgM, and IgA, and thyroid hormones (T3 and T4) were also determined. In addition, α-, β- and γ-globulin were measured according to a previously published method (51).

The contents of the duodenum, jejunum, and ileum were qualitatively collected and then kept in equal quantities of saline buffer. The mixture of each content was then centrifuged at 1,792 g for 15 min, and the supernatant was used to determine the levels of some digestive enzymes. The amylase activity was determined using the method described by Pinchasov et al. (51), lipase activity according to Sklan and Halevy (52), and trypsin and chymotrypsin activities according to Sklan et al. (53).

Representative samples of ilea, liver, thymus, and bursa (*n* = 5 per treatment) at 42 days of age were fastidiously dissected and placed in a sufficient volume of 10% buffered formal saline (BFS) for at least 24 h. The samples were prepared and measured as previously reported (54).

The proximate analyses of the experimental diets were performed according to the following official methods of analysis (55): dry matter, method number 934.01; crude protein, method number 954.01; ether extract, method number 920.39; crude fiber, method number 954.18; and ash, method number 942.05.

### Statistical Analysis

The data were analyzed with the Statistical Analysis System (SAS) software (56) using one-way analysis of variance (ANOVA) and the generalized linear model (GLM) procedure. The Student-Newman-Keuls test (56) was used to predict differences among the criteria; the effects were considered significant if *P* ≤ 0.05. All percentages were log base 10 transformed before ANOVA and then converted back to risk ratios for result presentation.

## Results and Discussion

### Growth Performance

Table 3 presents the effect of different oil sources on the performance of broilers during 1–42 days of age. Birds in this experiment show signs of thermal stress as evidenced by panting, lying on the floor and straightening the wing even the cooler group were absent, but heat stress behavior was evident Oil sources promoted positive effects on broiler chick growth during days 1–21 and 1–42 of age. The results also revealed that CocO source significantly increased chick growth during the 1–21-day period (9.9%) compared the FO-diet. However, Wang et al. (57) reported that a CocO-supplemented diet has no effect on weight gain. In addition, MCFA decreases the growth rate (35), and CocO enhances the digestion of fats and the performance index during coccidiosis infection (36).

**Table 3:** Effect of different dietary oil sources on growth performance of broiler chicks.

**Traits**	**Dietary treatments**				**RMSE**	***P*-values**
	**FO^1^-diet**	**CocO^2^ -diet**	**CanO^3^-diet**	**MTO^4^-diet**		
**Body weight**						
Initial 1 day, g	44.5	44.5	44.6	44.5	0.329	0.997
**Body weight gain**						
1–21days, g	537^b^	590^a^	583^a^^b^	575^a^^b^	28.6	0.022
1–42 days, g	2058^b^	2038^b^	2107^a^	2068^a^^b^	99.7	0.001
**Feed intake**						
21days, g	918^a^	853^b^	795^b^	845^b^	42.7	0.004
42 days, g	3290^a^	3097^b^	3020^b^	3156^a^^b^	115.9	0.015
**Feed conversion rate**						
21days, kg/kg	1.71^a^	1.45^b^^c^	1.37^c^	1.47^b^	0.061	0.001
42 days, kg/kg	1.60^a^	1.52^a^^b^	1.43^b^	1.53^a^^b^	0.075	0.022
**European production index**						
1–42 days	308	319	350	324	2.82	0.148
**Digestive enzymes activity, U/ml of intestinal content**						
Amylase	3.33	3.32	3.01	2.96	0.299	0.129
Lipase	11.7	12.7	12.8	12.5	1.65	0.712
Trypsin	28.2	28.9	28.4	24.8	2.65	0.089
Chymotrypsin	19.8	21.3	20.7	20.8	1.92	0.654

a,b,c Means for each trait with different superscripts differ significantly at p < 0.05;

1 FO, Fish oil,

2 CocO, coconut oil,

3 CanO, canola oil,

4 *MTO, mixture of three oils (FO + CocO + CanO)*.

For the whole period, CanO significantly elevated growth during days 1–42 compared to FO and CocO diets. The improved growth performance of broilers on CanO could be due to higher UFA and mono-unsaturated fatty acids (MUFA), and the higher energy capacity of PUFAs (23). The growth rate of female broilers fed two varieties of CanO is higher compared to broilers fed tallow and acidulated soybean oil soap stock. The increased growth is due to high LCFA and a high percentage of triglycerides (24).

In previous studies, fat-enhanced feeds elevate the efficiency of the consumed energy and productivity in poultry under normal and hot climate condition, but the impact depends on fat/oil source (2, 44, 45). Moreover, the oil improves the absorption of fat-soluble vitamins, the palatability of diets, decreases the dustiness of feeds and reduces the passage rate of feed in the gut, which allows more time for the sufficient absorption of nutrients particularly under high temperature due to impairing digestion of feeds (1–5, 44). For examples, Zollitsch et al. (39) and Khatun et al. (8) reported that broilers fed 6% soybean oil (mostly 84% an unsaturated oil) or a combination of soybean oil and palm oil (mostly 50% a saturated oil) exhibit significantly increased growth during days 1–21 of age compared to a control diet with 6% palm oil. Nobakht concluded that the growth rate of broiler chickens decreases with dietary FO inclusion (14). The contradiction among the above mentioned studies indicate that the impact of the type of fat on growth performance depends on the profile and levels of the utilized fatty acid(s) as well as the age of the chickens and type of stress (58). Chickens fed an erucic-acid-enriched diet exhibit a significantly lower growth rate, FI, and lipid and fatty acid digestibility (23) and store less fat and utilize energy less efficiency (23).

There was a significant effect on FI during days 1–21 and 1–42 (Table 3). FO significantly increased FI compared to other oils groups during days 1–21 of age. Furthermore, the FO diet increased FI by 8.9% compared to the CanO diet from days 1–42. The lowest FI was for CanO group from days 1–21 and 1–42 days of age. MTO supplementation presented an intermediate effect on FI. The FO-mediated FI increase compared to CocO, and CanO is in general agreement with Dawood and Mohammed (59). Besides, 3% palm oil significantly increases feed intake compared to soybean oil from days 17–38 of age but does not affect FI from days 1–16 of age (40). However, 6% palm oil or soybean oil or different combinations of the two does not affect feed intake of broilers during days 1–21 days and 22–42 age (8). According to the present findings, the addition of SFA increased the FI compared to UFA; this change depended on the level and source of fats and age of chickens. In the literature, the PUFA linolenic acid (18:3) and linoleic acid (18:2) decrease feed intake compared to SFA, i.e., palmitic and stearic acids (60). The decrease in FI observed herein of broilers fed CocO, and CanO might be due to PUFAs and their high energy-yielding capacity. In addition, the low digestibility of SFA compared to UFA was cited Zollitsch et al. (39) and Ayed et al. (40), particularly during the early weeks of life (3). However, Wang et al. (57) reported that broilers fed a CocO-supplemented diet showed no difference in FI compared to a FO-supplemented or control diet (14).

The broilers fed the CanO-supplemented diet from days 1–21 and 1–42 exhibited the best change in FCR compared to the other oil diet: 6.80–19.9% and 7.0–10.6%, respectively (Table 3). The worst FCR was from the FO-supplemented feeds from days 1–21 and 1–42 of age. Broilers fed the CocO- or MTO-enriched diet showed similar and better FCR than the FO during days 1–21 (15.2%). The present results indicate that CanO, which is rich in linolenic and linoleic acids, enhances broiler growth and FCR due to its high energy-yielding capacity compared to diets rich in SFA. These enhancements were associated with increased growth. The results also demonstrate that CanO used herein had low erucic acid content and thus had no harmful effect on broilers (23, 25). Likewise, oil supplementations improved FCR of broiler under normal and high ambient temperature (2, 44, 45). In literature, differences in FCR due to various oil sources are exist for example, Wignjosoesastro et al. (61) revealed that 10% CocO increases the rate of production and efficiency. In addition, Zollitsch et al. (39) and Ayed et al. (40) observed that broilers fed soybean oil present a significantly improved FCR compared and palm oil diets. Besides, Khatun et al. (8) revealed that a 6% PUFA-supplemented diet and a combination of soybean and palm oils significantly enhance the FCR compared to the 6% palm oil diet. However, broilers fed sunflower oil have a better FCR compared to those fed beef tallow (61). On the other hand, Wang et al. (57) reported that broilers fed a CocO-enriched diet exhibit no difference in FCR compared to control. Nobakht (14) concluded that the dietary FO level does not affect the FCR of broilers.

There were no significant differences in European production Index and mortality under the present experiment condition.

### Digestive Enzyme Activity

The activity of the digestive enzymes in the gut (proventriculus, duodenum, jejunum, and ileum) as influenced by different dietary oil sources is shown in Table 3. The measurements estimated at day 42 of age after exposure of broilers to high ambient temperature during 4–6 weeks of age indicate that there were no significant differences due to different oil sources on digestive enzyme activates. The inclusion of FO, and CocO numerically increased intestinal amylase (10.5–12.5%) compared to CanO and the combination of all three oils. In addition, CocO-, CanO-, and MTO enriched diets numerically elevated intestinal lipase 6.8–9.4% compared to the FO-supplemented diet. However, there were a trend (*P* < 0.089) for trypsin to be numerically higher (16.1%) in the groups that received the individual fat sources compared to the MTO group. Chymotrypsin activities numerically also increased (2.5%) due to CocO compared to FO. A previous study indicated higher activities of pancreatic trypsin, α-amylase, and intestinal maltase due to oil supplementation (62). In general, the improved digestive enzyme activities found herein are consistent with the increased growth and feed use for growth under hot conditions (Table 3). Along the same line, CocO improves fat digestion and performance values during coccidiosis infection (36).

### Carcass Characteristics

The dressing, liver, gizzard, heart, giblets, total edible part, and abdominal fat after supplementation with the different oil sources are shown in Table 4. The parameters measured at day 42 of age indicate that broilers fed the CocO-, CanO-, and MTO-enriched diets had a similar dressing percentage, and a resulted in 2.8–4.5% increase in total edible parts compared to the FO-supplemented feeds. Moreover, MTO supplementation had no additive effects on carcass parameters and organs traits compared to induvial oil sources. This indicates that individual supplementation of oil source was adequate, and oil supplementations during hot weather condition are beneficial (2, 44, 45). Broilers fed the CocO, CanO-, and MTO-enriched diets had lower abdominal fat percentage (32%) compared to the FO diets, but the liver percentage was significantly higher (22.9%) with the CanO-enriched diet compared to the CocO. The increased dressing and total edible parts of broilers fed a PUFA-enriched diet (CocO, CanO, or MTO) indicates that these animals had higher energy availability for muscle growth, while the decrease in abdominal fat in these groups shows a shift in energy use for muscle growth rather than deposition in the abdominal cavity (14, 15). In addition, Baião and Lara (3) and Nobakht et al. (14); demonstrated that the inclusion of different PUFA sources and levels in broiler diets significantly influenced carcass traits. Moreover, including 5% CanO in broiler diets increases the breast weight compared to the other groups (63). Similar performance and carcass and cut yields were reported for broilers fed different sources of fats (2, 25, 44, 45). The improved carcass yield and the decrease in abdominal fat and liver percentage of broilers fed the CocO-enriched diet indicates that oil is burnt for energy rather than stored in the body (27). Consumption of a feed rich in MCFA increases upper body adiposity in overweight men; hence, MCFA may be considered as a potential tool in the prevention of weight gain and obesity (27, 32). Consistent with the reduction in the dressing and total edible parts of broilers, the FO-supplemented diet negatively affected carcass criteria (4, 64).

**Table 4:** Effect of feeding different oil sources on carcass characteristics at 42 days (%).

**Traits**	**Dietary treatments**				**RMSE**	***P*-values**
	**FO^1^-diet**	**CocO ^2^-diet**	**CanO^3^-diet**	**MTO^4^ -diet**		
Dressing, %	71.3^c^	73.6^b^	74.6 ^a^	74.9^a^	0.626	0.001
Liver, %	2.52^a^^b^	2.31^b^	2.84^a^	2.54^a^^b^	0.266	0.049
Gizzard, %	2.61	2.67	2.59	2.57	0.354	0.969
Heart, %	0.569	0.549	0.516	0.560	0.063	0.579
Giblets, %	5.70	5.53	5.94	5.67	0.320	0.280
Total edible part, %	77.0^c^	79.2^b^	80.5^a^	80.2^a^	0.725	0.001
Abdominal fat, %	2.42^a^	1.77^b^	1.73^b^	1.71^b^	0.261	0.002

a,b,c Means for each trait with different superscripts differ significantly at p < 0.05;

1 FO, Fish oil,

2 CocO, coconut oil,

3 CanO, canola oil,

4 *MTO, mixture of three oils (FO + CocO + CanO)*.

The observed increase in the liver percentage of the broilers fed the CanO-enriched diet was due to additional fat deposition in this organ rather than as abdominal fat. This phenomenon is likely due to the higher UFA content in CanO (64.8%), of which oleic is dominant. The ME of oils and fats depends on the number of double bonds, the length of the carbon chain, the presence or absence of ester bonds (free fatty acids or triglycerides), the dietary composition (3). In addition, the specific configuration of the UFA and SFA in the glycerol backbone, the free fatty acid arrangement, the amount and the kind of the nutritional triglyceride contents, the gut microflora, and the age and the sex of the chicken (3, 39).

### Plasma Lipid Profile

Table 5 presents the influence of different oil sources on the plasma lipid profile of 42-day-old broilers. Plasma triglycerides, total cholesterol, LDL-C, HDL-C, and the LDL-C:HDL-C ratio and vLDL of broilers fed diets with different sources of oils were similar. The total plasma lipids was significantly higher in the FO and CanO groups compared to the MOT group, which showed the greatest decrease (15.5%) in plasma total lipids. The CocO groups presented intermediate values. The plasma concentrations of triglycerides/HDL showed a trend toward (*P* < 0.093) increased values (10.5–12.6%) of CanO and MTO groups compared to FO and CocO groups. Overall, the change in plasma lipid profiles, reflecting the dietary fatty acid composition and correlated with the increased dietary PUFA content, as well as elevated intestinal lipase activity. The positive effect of CocO supplementation found herein on the plasma total lipids, triglycerides/HDL indicates that oil is burnt for energy and use for dissipate heat stress (27, 32, 44, 45). Similarly, the MCFA improve serum lipid profiles in humans and rats (31, 34). Researchers demonstrated that UFA and beneficial PUFA have positive and healthy influences on plasma lipids due to its impact on increasing HDL-C while decreasing LDL-C, the hazardous lipoprotein segment (61, 65–67).

**Table 5:** Effect of feeding different oil sources on some blood plasma lipid biochemical constituents; antioxidants indices; thyroid hormones and liver leakage indices.

**Traits**	**Dietary treatments**				**RMSE**	***P*-values**
	**FO^1^-diet**	**CocO^2^-diet**	**CanO^3^-diet**	**MTO^4^-diet**		
**Plasma lipid metabolites**						
Total lipids, mg/dl	560^a^	514^a^^b^	576^a^	487^b^	41.9	0.016
Triglycerides, mg/dl	144	144	150	157	10.3	0.206
Cholesterol, mg/dl	169	172	168	173	11.3	0.632
HDL, mg/dl	65.5	65.8	62.8	62.7	6.18	0.782
Triglycerides:HDL ratio	2.22	2.19	2.42	2.50	0.213	0.093
LDL, mg/dl	74.7	77.8	75.2	79.1	11.3	0.912
LDL: HDL ratio	1.17	1.20	1.21	1.26	0.258	0.948
vLDL, mg/dl	28.9	28.8	30.1	31.4	2.05	0.206
**Plasma thyroid hormones**						
T4, ng/Ml	20.6	22.1	21.2	23.6	1.95	0.131
T3, ng/mL	4.51^b^	5.18^a^	4.59^b^	5.23 ^a^	0.345	0.006
T3:T4 ratio	0.219	0.236	0.217	0.223	0.018	0.354
**Plasma antioxidant enzymes**						
CAT, U/ml/h	61.5 ^b^	74.1^a^	68.1^a^^b^	67.2^a^^b^	5.08	0.012
SOD, U/ml/h	75.5^b^	88.7^a^	84.2^a^	91.1^a^	4.89	0.001
TAC, nmol/ml	1.21^b^	1.36 ^a^	1.40^a^	1.34^a^	0.091	0.023
MDA, nmol/ml	22.2^a^	17.2^b^	21.2^a^^b^	17.6^b^	2.69	0.021
MDA:TAC ratio	18.5^a^	12.7^b^	15.2^b^	13.1^b^	2.02	0.002
**Plasma liver leakage indices**						
AST, U/dL	68.2^b^	57.9^c^	78.7^a^	65.7^b^	4.25	0.001
ALT, U/dL	26.1^a^	18.8^b^	24.3^a^	24.1^a^	1.99	0.001
AlkP, U/dL	56.6^a^	43.3^b^	65.4^a^	64.1^a^	5.75	0.001

a,b,c Means for each trait with different superscripts differ significantly at p < 0.05; HDL, High density lipoprotein; LDL, Low density lipoprotein; vLDL, Very low density lipoprotein; CAT, Catalase; SOD, super oxide dismutase; TAC, Total antioxidant capacity; MDA, Malondialdehyde; T4, Thyroxine; T3, Triiodothyronine; AST, Aspartate amino transferase; ALT, Alanine amino transferase;

1 FO, Fish oil;

2 CocO, coconut oil;

3 CanO, canola oil;

4 *MTO, mixture of three oils (FO + CocO + CanO)*.

### Plasma Thyroid Hormones

Thyroid hormones as affected by different types of oils are shown in Table 6. The results show that CocO and MTO supplementation promoted the greatest increases in T_3_ compared to the FO and CanO diets. On the other hand, T_4_ and T_3_:T_4_ ratio were not affected by dietary fat sources. Thyroid hormones decreased during hot weather and are involved in the regulation of anabolic and catabolic pathways of protein, lipid and carbohydrate metabolism (68, 69). A previous study reported that a significant interaction between the type and level of fats might have adverse effects on T_3_ and T_4_ levels (68, 69). The dietary fatty acid profile reportedly affects deiodinase hepatic type I activity (68, 69) and binding of T_3_ to nuclear receptors (70). This finding partially contradicts the results of research that showed a reduction in T_3_ level of animals fed high-fat diets (71, 72). However, it is difficult to explain the lack of effect of fat level on the T_3_ concentration in rats fed palm and rapeseed oils.

**Table 6:** Effect of feeding different oil sources on blood serum immune indices, and morphometric characteristics of Intestinal villi, bursa of Fabricius and thymus cortex.

**Triats**	**Dietary treatments**				**RMSE**	***P*-values**
	**FO^1^-diet**	**CocO ^2^-diet**	**CanO^3^-diet**	**MTO^4^-diet**		
**Serum protein metabolites**						
Total serum protein, g/dl	3.17^a^	2.14^c^	3.14^a^	2.64^b^	0.226	0.001
Albumin, %	32.2^b^	46.8^a^	52.3^a^	45.9^a^	7.07	0.003
α1-globulin, %	1.78^b^	8.48^a^	7.47^a^	3.70^b^	2.43	0.002
α2-globulin, %	5.84	3.87	5.55	6.17	1.91	0.268
β-globulin, %	8.92^a^	4.88^b^	9.33^a^	5.57^b^	2.25	0.012
γ-globulin, %	42.6^a^^b^	24.0^c^	35.9^b^	47.9^a^	6.09	0.001
A:G ratio	1.20	1.34	1.40	1.26	0.192	0.405
**Serum immunoglobulin**						
IgG, mg/dL	460^b^	541^a^	542^a^	572^a^	23.9	0.001
IgM, mg/dL	191^b^	242^a^	253^a^	244^a^	18.9	0.001
IgA, mg/dL	127	137	133	138	13.9	0.636
**Serum antibody titer**						
HIBB, log^2^	4.24	3.62	3.23	4.21	1.08	0.718
HIBD, log^2^	3.62	2.89	3.11	3.34	1.14	0.451
HIAI, log^2^	3.66^a^^b^	4.23^a^	3.12^b^	3.17^b^	0.510	0.005
HIND, log^2^	5.61^b^	7.02^a^	6.23^a^^b^	6.07^a^^b^	0.688	0.037
**Intestinal villi parameters**						
Ville height, μm	623^b^	522^c^	661^a^^b^	703^a^	40.2	0.001
Crypt depth, μm	106	112	109	99.1	10.9	0.320
Ville height:depth ratio	5.94^a^	4.76^b^	6.19^a^	7.18^a^	0.763	0.001
**Bursa of Fabricius follicle and thymus characteristics**						
Follicle length, μm	471^b^	474^b^	506^a^	512^a^	20.5	0.009
Follicle width, μm	355^a^	310^a^^b^	278^b^	304^a^^b^	36.5	0.033
Length: width ratio	1.34^b^	1.56^a^^b^	1.82^a^	1.70^a^	0.185	0.006
Thymus cortex depth, μm	112^b^	123^b^	155^a^	129^b^	13.93	0.001

a,b,c Means for each trait with different superscripts differ significantly at p < 0.05; α1-globulin, Alpha 1-globulin; α2-globulin, Aalpha 2 globulin; β-globulin, Beta-globulin; γ-globulin, Gamma-globulin; IgG, Immunoglobulin G; IgM, Immunoglobulin M; IgA, Immunoglobulin A; HIBB, Hemagglutination-inhibition test for infectious bronchitis virus; HIBD, Hemagglutination-inhibition test for bursa disease; HIAI, Hemagglutination-inhibition test for avian Influenza; HIND, Hemagglutination-inhibition test for Newcastle disease virus;

1 FO, Fish oil;

2 CocO, coconut oil;

3 CanO, canola oil;

4 *MTO, mixture of three oils (FO + CocO + CanO)*.

The increase in T_3_ of broilers supplemented with CocO diets under hot climate condition may be due to the elevated energy availability and use for an anabolic processes for muscle growth. It is evidenced by the rise in the growth rate and FCR during 1–21 days of and the decrease in liver and fat deposition in the abdominal cavity of broilers fed a CocO-supplemented diet (Table 4). CocO is a rich source of SFA and MCFA (6–12 carbon atoms) (3, 27), which can be absorbed directly into the portal system without re-esterification in intestinal cells (28). MCFA are exclusively and rapidly burned to produce energy (29). By contrast, LCFA is commonly found in most diets and are incorporated into chylomicrons after being absorbed in the intestine, where they are subjected to re-esterification, and then reach the bloodstream via the lymphatic system (28). Most LCFA are stored in adipose tissue (30). In one study, the total T_4_ levels are higher in Wistar rats fed 5 or 10% palm-oil-enriched compared with a rapeseed-oil-enriched diet (68).

### Plasma Antioxidant Status

The supplementation with CocO, CanO, or MTO significantly—and similarly—increased SOD, and TAC activities compared to the FO group (Table 6). In addition, CocO significantly increased CAT compared to the FO. Besides, CocO- and MOT-supplemented diets significantly decreased plasma MDA and the MDA: TAC ratio compared to the FO diet. In addition, CanO substantially reduced MDA: TAC ratio compared to FO group, and MAD to some extent. In general, CocO supplementation induced the most substantial effect on the antioxidant profiles, while FO enrichment promoted the smallest influence. It is well known that MDA increased due feeding PUFA and particularly during hot weather condition due to peroxidation process (44). This beneficial effect of oils on the antioxidant profile shows the essence of oil supplementation during hot weather condition, particularly when a combined with antioxidant supplementation (45, 65). Notably, Bhatnagar et al. (27) reported that CocO dietary supplementation increases total tocopherols. Tocopherols are essential antioxidants that protect the cell membrane from free radicals (73). Furthermore, CocO is very stable against oxidation and, consequently, not prone to peroxide formation. Therefore, the incorporation of CocO enhances the constancy of the feeds (27). Besides, CocO has unique antibacterial, antiprotozoal, and antiviral effects, all of which control microbial rancidity (74, 75).

### Hepatic Plasma Leakage Enzymes

Table 6 also shows the effect of different oil sources on the plasma hepatic leakage enzymes of 42-day-old broilers. AST, ALT, and AlkP were significantly lower in chickens fed the CocO-enriched diet compared to those of the other oil groups. Furthermore, broiler chicks fed FO and MTO presented considerably decreased plasma AST activity compared to the CanO groups and raised AlkP compared to the CocO group. These results indicate a beneficial effect of PUFA on hepatic cell membrane integrity that might be due to enriched phospholipids as an essential part of cell membrane integrity containing two hydrophobic LCFA (4, 5, 65). The beneficial effects of CocO on liver leakage enzymes as previously said might be due to its antioxidant (27) and antimicrobial effects (74, 75). In literature, oil supplementation improved liver function (44, 45). However, Yildirim et al. (66) found that dietary fat sources, including cocoa butter, did not affect plasma AST, ALT and AlkP in rats. The differences among the studies might be due to the type of diets, levels and the kind of lipids, strain, age and sex, and species of animals (73).

### Immune Status

#### Serum Protein Profile

Table 6 also shows the influence of different oil sources on serum immune indices of the broilers. The different oil sources had no effect on α2-globulin or albumin to globulin ratio measured at day 42 of age. The FO- and CanO-supplemented diets similarly increased the total serum protein compared to the CocO and MTO group. In general, the improvement in protein use with FO and CanO supplementation found herein can enhance the immune response, and the antibodies are proteinic in nature. The increased serum protein of broilers fed the FO- or CanO-enriched diet are in general agreement with the effect of oils on improving nutrient absorption (including protein) under hot condition (3, 4, 45). The present results confirmed this effect because the different FO, and CanO oil sources increased chymotrypsin activity. The increased enzyme activity is associated with raising nutrient digestibility and thus, nutrient absorption (1, 44, 65).

The FO-supplemented diet significantly decreased the percentage of albumin (a non-specific immune protein) compared to the other oil groups. Likewise, FO supplementation decreased T- and B-cell proliferation and delayed type hypersensitivity as measurements of cell-mediated immunity (76, 77).

#### Serum Immunoglobulin

The CocO-, and CanO-enriched diets significantly increased α1-globulin compared to the FO and MTO groups. The difference in β-globulin due to the different oil sources showed that FO and CanO induced substernal increase compared to CocO and MTO. The γ-globulin was raised considerably by MTO-enriched diet compared to the CocO, and CanO- enriched groups. Immunoglobulins (γ-globulin) play a vital role in natural and acquired immunity to infections (78). Globulins—a major family of proteins—are an essential source of the protein present in animal fluids, including the enzymes, antibodies, and fibrous and contractile proteins usually found in the blood plasma (73). α-and β-globulins are transport proteins, serve as substrates upon which other substances are formed, and perform other diverse functions (78).

Different oil sources did not meaningfully affect IgA, but IgG and IgM were decreased considerably due to FO supplementation compared to the other oil groups. However, Yildirim et al. (66) revealed no marked changes in IgG among their experimental groups. Besides, the IgM levels were significantly reduced in cocoa butter alone and cocoa butter + sunflower oil group as compared to the sunflower-oil-enriched diet and the control group.

#### Serum Antibody Titer

None of the oil sources affected the HI test for infectious bronchitis virus (HIBB) or bursa disease (HIBD) (Table 6). On the other hand, broilers fed a diet supplemented with 50 g/kg FO show a higher production of antibodies (IgM and IgG) and globulins in the serum and maintain immune function after vaccination compared to the control group (79). In the present study, the addition of CocO resulted in a considerable increase in the HIAI compared to the CanO and MTO groups. In addition, the CocO-supplemented group exhibited significantly increased HIND compared to the FO-group. In general, CocO induced the highest antibody titers to HIAI and HIND. MCFA can be absorbed through the portal circulation after ingestion. Subsequently, they are metabolized by hepatocytes into ketones and used as an energy source. This process maintains the serum leptin level and promotes ketogenesis during short-term fasting and energy restriction (80). Leptin can enhance immune functions, including inflammatory cytokine production in macrophages, granulocyte chemotaxis, and increased Th17 proliferation (81). The ketogenic diet activates a subset of T cells in the lungs not previously associated with the immune system's response to influenza; this action enhances mucus production by airway cells that can effectively trap the virus (82). Nonetheless, the level and source of dietary fat did not significantly influence antibody titer against NDV at 42 and 70 days of age (80, 83), but had a positive effect on antibody in other experiments (44, 45). In humans, dietary CocO normalize body lipids, protect the liver from alcohol damage, and improve the immune system's anti-inflammatory response (27, 74, 75). Similarly, Yaqoob (12) suggested that the impacts of MUFA on adhesion molecules are potentially crucial, namely through their roles in the pathology of several diseases involving the immune system. There is some evidence that the olive oil effects on immune function in animal studies are due to oleic acid rather than to trace elements or antioxidants. Kelley and Daudu (84) reported that in animals fats could inhibit and stimulate processes, depending upon the species, the utilized fatty acids, and the index being examined. These findings suggest that the absolute amounts or the ratios between individual fatty acids or fatty acid classes are critical in determining their effects on the immune response and need to be investigated (12, 83).

It is worth noticing that, FO supplementation resulting in increasing HIAI numerically and correlated with an increase in γ-globin and a decrease in serum albumin. Dietary ω3 PUFA (FO or linseed oil) has a positive significant impact on humoral immunity, i.e., antibody titers against NDV compared to the control diet (18). Furthermore, FO supplementation significantly increases antibody titers and the relative weight percentages of the bursa and spleen compared to the control under normal and hot climate condition (20, 44, 45). FO has no adverse effects on the immune function of broilers (19). On the other hand, birds fed a diet with an ω6: ω3 ratio that exceeds the recommended levels exhibit damage to the intestinal epithelial cells (21). Further, a low ω6: ω3 ratio in the diet increases MDA in tissues, including the meat, and thus impairs meat quality.

### Morphometric Measurements of the Villi, Bursa of Fabricius and Thymus

The villus height and villus height: depth ratio were significantly affected by dietary oil supplementation (Table 6). The MTO significantly increased the villus height compared to the FO and CocO groups, and increased the villus height: depth ratio compared to CocO group. These results indicate that FO, CanO and MTO increase the absorption capacity of the intestine and thus might be responsible for improving the growth and immunity of broilers fed these diets. This is an essential influence during hot weather condition due to impaired digestion and absorption of nutrients (44, 45, 65, 73).

Follicle parameters and thymus cortex depth were significantly affected by the source of oils. The addition of CanO and MTO caused the highest increase in follicle length compared to the other sources of oil, but FO significantly increased the follicle width compared to the CanO group. The follicle length: width ratio was significantly decreased in broilers fed the FO-enriched diet compared to the CanO and MTO groups.

The thymus cortex depth was significantly increased for CocO-supplemented diets compared with the other oil groups. Similarly, the source of fat had significant effects on the weight and percentage of thymus and ratio of the bursa of the Fabricius to body weight (4, 5). Nonetheless, the weight of the bursa of Fabricius and weight and percentage of the spleen, humoral immune response to SRBC injections or to NDV, IBDV or IBV vaccinations were not affected by oil treatments (4, 5). The addition of 2.5 or 5% soybean oil to the diet increases the percentage of spleen and bursa, respectively, compared to other groups (83). Taken together, the fatty acid profile rather than the specific dietary lipid is essential to optimize cellular and humoral immunity in broilers (84).

In conclusion, oil supplementations at 1.5% enhanced growth performance and immune status, improved the blood lipid profile and antioxidants status, and the effect of the oil sources depends on the criteria of response.

## Data Availability Statement

The datasets generated for this study are available on request to the corresponding author.

## Ethics Statement

The experimental procedures were approved by Deanship of Scientific Research, King Abdulaziz University, Jeddah, Saudi Arabia under protocol number (DF-312-155-1441 H) that recommends animal rights, welfare and minimal stress and did not cause any harm or suffering to animals according to the Royal Decree number M59 in 14/9/1431H.

## Author Contributions

All authors listed have made a substantial, direct and intellectual contribution to the work, and approved it for publication.

## Conflict of Interest

The authors declare that the research was conducted in the absence of any commercial or financial relationships that could be construed as a potential conflict of interest.
